# Assessment of *Babesia bovis* 6cys A and 6cys B as components of transmission blocking vaccines for babesiosis

**DOI:** 10.1186/s13071-021-04712-7

**Published:** 2021-04-20

**Authors:** Heba F. Alzan, Reginaldo G. Bastos, Massaro W. Ueti, Jacob M. Laughery, Vignesh A. Rathinasamy, Brian M. Cooke, Carlos E. Suarez

**Affiliations:** 1 Department of Veterinary Microbiology and Pathology, College of Veterinary Medicine, Washington State University, Pullman, WA USA; 2 Parasitology and Animal Diseases Department, National Research Center, Dokki, Giza, Egypt; 3 Tick and Tick-Borne Disease Research Unit, National Research Center, Dokki, Giza, 12622 Egypt; 4 Animal Disease Research Unit, United States Department of Agricultural - Agricultural Research Service, Pullman, WA USA; 5 Australian Institute of Tropical Health and Medicine, James Cook University, Cairns, Queensland Australia

**Keywords:** *Babesia bovis*, Transmission blocking vaccine, Tick, *Rhipicephalus microplus*, Sexual stages, Synthetic peptides, Recombinant proteins, Neutralizing antibodies

## Abstract

**Background:**

*Babesia bovis* reproduces sexually in the gut of its tick vector *Rhipicephalus microplus*, which involves expression of 6cys A and 6cys B proteins. Members of the widely conserved 6cys superfamily are candidates for transmission blocking vaccines (TBV), but intricacies in the immunogenicity of the 6cys proteins in the related *Plasmodium* parasites required the identification of transmission blocking domains in these molecules for vaccine design. Hereby, the immunogenic efficacy of recombinant (r) *B. bovis* 6cys A and B proteins as a TBV formulation was studied.

**Methods:**

The immunogenicity of r6cys A and 6cys B proteins expressed in a eukaryotic system was evaluated in a cattle immunization trial (3 immunized and 3 control calves). A *B. bovis* sexual stage induction *in vitro* inhibition assay to assess the ability of antibodies to block the production of sexual forms by the parasite was developed.

**Results:**

Immunized cattle generated antibodies against r6cys A and r6cys B that were unable to block sexual reproduction of the parasite in ticks. Additionally, these antibodies also failed in recognizing native 6cys A and 6cys B and peptides representing 6cys A and 6cys B functional domains and in inhibiting the development of sexual forms in an *in vitro* induction system. In contrast, rabbit antibodies generated against synthetic peptides representing predicted B-cell epitopes of 6cys A and 6cys B recognized recombinant and native forms of both 6cys proteins as well as peptides representing 6cys A and 6cys B functional domains and were able to neutralize development of sexual forms of the parasite *in vitro*.

**Conclusions:**

These data, combined with similar work performed on *Plasmodium* 6cys proteins, indicate that an effective 6cys protein-based TBV against *B. bovis* will require identifying and targeting selected regions of proteins containing epitopes able to reduce transmission.

**Graphic abstract:**

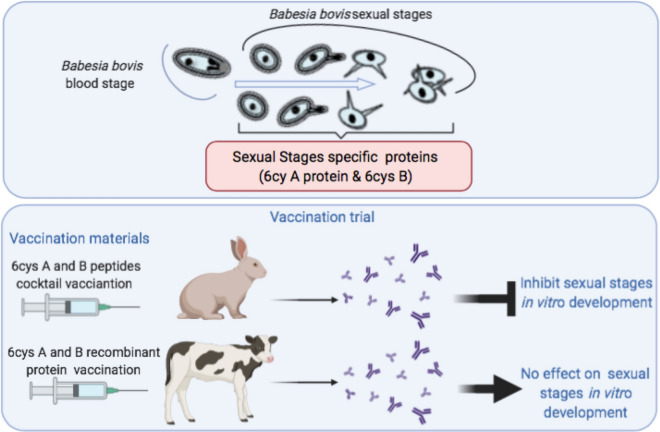

**Supplementary Information:**

The online version contains supplementary material available at 10.1186/s13071-021-04712-7.

## Background

Bovine babesiosis is a tick-transmitted protozoan parasitic disease of cattle with global impact and is mainly caused by *Babesia bovis, B. bigemina* and *B. divergens* [1]. The disease causes major economic losses to the cattle industry in tropical and sub-tropical regions worldwide. Current control strategies for bovine babesiosis are based on tick control, use of anti-*Babesia* drugs and live-attenuated vaccines, but these approaches have numerous limitations [2–5]. The intensive use of acaricidal drugs can result in enzootic instability, environmental damage and the development of acaricide-resistant ticks [6]. Similarly, current anti-*Babesia* drugs pose the risk of inducing the development of drug-resistant parasite strains, and residues may be present in meat and milk products, which prohibits their use in some countries [7]. Although the currently available live-attenuated *B. bovis* vaccines have proved to be effective in several countries that permit their use, critical drawbacks remain. These limitations include the risk of reversion to virulence of the parasites in the vaccine, contamination with other pathogens during passage through live splenectomized calves for attenuation and the need for a cold chain for transportation and delivery [8, 9]. Importantly, these vaccines only target blood-stage *B. bovis* parasites and can only be effective when given to young calves (< 1 year old) [2–5]. Therefore, effective control of bovine babesiosis requires the development of new alternative vaccines, including transmission blocking vaccines (TBVs). This can limit expansion of the parasite by ticks in *Babesia*-endemic areas, while avoiding the current drawbacks of current live vaccines.

Following a similar rationale for *Plasmodium* TBVs, TBVs against *B. bovis* can be designed to elicit immune responses in vaccinated cattle and consequently interfere with the development of parasite sexual stages inside the tick vector. Eventually this can arrest the parasite life cycle in the ticks, leading to the suppression of transovarial transmission and potentially to the eradication of the disease [10, 11]. However, a control approach based solely on TBVs might not be fully effective in areas where cattle co-exist with wild fauna infested with *Babesia*-infected ticks. Therefore, and considering that TBVs are not designed to interfere with development of the parasite in the vertebrate host, they could be combined with vaccine antigens from blood-stage parasites that may help to ameliorate acute disease, which might increase their utility.

We previously identified and characterized the 6cys A and 6cys B proteins of *B. bovis* and showed that these proteins are markers for sexual stage development inside the tick host [12, 13]. Moreover, limited polymorphism, combined with high conservation among distinct *B*. *bovis* strains, and the lack of their expression in blood stage parasites [12] are two strong appealing properties of 6cys A and 6cys B proteins as potential TBV antigens. High levels of sequence conservation and tick-stage specific expression suggest that these 6cys proteins are required to perform key biological functions during parasite development in the tick vector. Therefore, they are potential targets for TBVs that could likely induce effective antibody responses against heterologous *B. bovis* strains, by interfering with parasite reproduction in ticks.

Several mosquito-stage-specific proteins, including members of the 6cys superfamily P25, P28, P230, P48/45 and Pfs47, have been tested as TBVs and showed significant transmission-blocking activity against *Plasmodium* [10, 11, 14]. It remains unknown whether this is also the case for the *B. bovis* 6cys proteins.

Hereby, we tested whether bovine antibodies against full-length recombinant 6cys A and 6cys B proteins interfere with sexual stage parasite development and sexual reproduction of *B. bovis* within *Rhipicephalus microplus* ticks. In addition, we compared bovine antibodies with rabbit polyclonal antibodies against selected regions of 6cys A and 6cys B containing predicted B-cell epitopes [12] in their ability to recognize native 6cys proteins and to inhibit the development of sexual forms in an *in vitro* sexual stage induction system. Overall, results of this study support the relevance of these 6cys proteins for sexual stage development and highlight the notion that an effective TBV based on the 6cys antigens will likely require the identification of transmission-reduction (TR) epitopes, which could be able to elicit antibodies that effectively inhibit transmission of *B. bovis* via competent ticks.

## Methods

### Antigen preparation

Recombinant proteins including full-length [amino acid residues 1–598] *B. bovis* 6cys A (r6cys A) and full-length [amino acid residues 1–594] 6cys B (r6cys B) were expressed as 6x histidine-tagged proteins in a eukaryotic expression vector (pTT5) transfected in mammalian human embryonic kidney (HEK) cells (293–6E cells) and purified by HisTrap FF Crude histidine-tagged protein purification columns (GenScript, NJ, USA). The recombinant protein production was confirmed with anti-His antibodies (Additional file 2: Fig. S1). Antigenicity of r6cys A and r6cys B was tested using serum from *B. bovis* (T2Bo) infected cattle or polyclonal antibodies from rabbits immunized with cocktails of either 6cys A or 6cys B synthetic peptides predicted to contain B-cell epitopes [12]. The peptide cocktail 6cys A contains the synthetic peptides 6cys A1, A2 and A3, and peptide cocktail 6cys B includes the synthetic peptides 6cys B1 and B2, which we have previously described [12]. All proteins and peptides were stored at − 20 °C or − 80 °C until use. Cryopreserved *B. bovis* T2Bo parasite stabilates prepared from an infected calf and stored in liquid nitrogen were used as the source of infectious material for experimental infections [15].

### Immunization of calves

Six *Bos taurus* (Holstein breed) male calves, 3 to 4 months old at the beginning of the experiment, were used in the immunization trial. Two groups of spleen-intact calves were immunized four times subcutaneously at 21-day intervals. Group 1, comprising three calves [C1501, C1506 and C1508], was inoculated with a combination of *B. bovis* r6cys A and r6cys B antigens in separate inoculums, with each single recombinant protein inoculated at different sites. The r6cys A was applied at the right side and the r6cys B at the left side of the neck in each calf as 1 ml inoculum for each protein. The same sides were used for each individual protein in every immunization. The recombinant 6cys proteins were suspended in saponin adjuvant (lot 115H0913, Sigma, Germany). Group 2 (adjuvant control) comprised three calves, [C1502, C1505 and C1512] that were inoculated with saponin adjuvant alone in PBS (Additional file 3: Fig. S2). Each calf in the immunized group received 50 μg of r6cys A and 50 μg of r6cys B diluted in PBS and mixed with adjuvant while calves in the control group received 1 ml of adjuvant only per inoculation. All calves were closely monitored after each immunization for localized inflammatory responses at the injection sites and any signs of discomfort. Levels of immunoglobulin (Ig) G, IgG1 and IgG2 against *B. bovis* r6cys A and r6cys B were analyzed by indirect ELISA using serum samples collected before and after immunization at different time points throughout the trial.

### Tick infestation, *B. bovis* challenge, and tick and larvae analysis

One quarter of a gram of *R. microplus* larvae (La Minita strain) [16] was applied under a cloth on the six animals 21 days after the last immunization. When *R. microplus* nymphs began molting to adults (~ 14 days), calves were inoculated intravenously with ~ 10^7^ bovine red blood cells infected with *B. bovis* (T2Bo strain) to synchronize female tick engorgement with peak parasitemia in the animal. Animals were monitored daily for the signs of acute *B. bovis* infection, including fever, parasitemia (assessed by Giemsa-stained peripheral blood smears) and reduction in packed cell volume (PCV). Blood samples were collected daily from all six calves for 12 days post-infection (PI) and tested for the presence of *B. bovis* DNA by conventional PCR targeting the rap-1 gene [15] and qPCR using primers targeting the msa-1 gene [17].

The number of engorged ticks dropping from animals each day was counted manually. Weights of engorged ticks and egg mass, hatching rate, larval viability and levels of *B. bovis* infection in the offspring larvae were estimated from both immunized and adjuvant-control groups (Additional file 3: Fig. S2). Engorged female ticks were collected daily from day 8 to day 12 *B. bovis* PI, a time that coincided with peak parasitemia in the animals. Ticks collected from individual animal per day were pooled together and assigned to one group. In total, we collected five groups of ticks from each animal in each treatment group (immunized and adjuvant control) from day 8 to day 12 PI. Collected ticks were rinsed in tap water, placed into individual wells in 24-well tissue culture plates and incubated at 26 °C with 96% relative humidity to stimulate *B. bovis* tick-stage development, as previously described [16].

Hemolymph obtained from 48 engorged ticks per calf from each group (immunized and control) was evaluated for the presence of *B. bovis* kinetes. Briefly, the distal leg segment was removed from each individual tick, and exuding drops of hemolymph were blotted on glass slides which were stained with the Giemsa stain. The stained slides were examined by light microscopy (for all five tick groups) [16]. Also, the hemolymph was tested by qPCR (only for tick groups 4 and 5, with the highest infection rate) for the presence of *B. bovis* DNA. In addition, hemolymph from 48 individual engorged and dropped female ticks per calf was collected on day 4 and day 7 days post repletion and used to determine the presence of bovine antibodies against the r6cys A and r6cys B proteins. A total of 96 random dropped female ticks per day, for 5 consecutive days per calf, were weighed individually, and the average weights were calculated and compared between immunized and control groups. Pooled eggs from 48 ticks per animal per group were weighed and compared between immunized and control groups.

The presence of *B. bovis* DNA in offspring larvae fed on both immunized and control animals was also investigated by qPCR. Tick larvae were incubated at 35 °C and 26 °C and analyzed for the presence of *B. bovis* DNA 82 days after hatching. A total of 100 larvae were tested from each group (Additional file 4: Fig. S3). The paired Student *t*-test was used to compare experimental groups.

We used Fisher’s exact test to determine the minimum number of ticks required for analyzing the numbers of *B. bovis* kinetes circulating in hemolymph with significant statistical power. Collecting at least 14 ticks per animal per day for analysis provides 90% power. Based on this calculation, we decided to analyze 48 ticks per calf.

### Serological analyses

Recombinant antigen-specific antibody responses (total IgG, IgG1 and IgG2) in immunized and control animals were determined by indirect ELISA using serum samples collected from day 0 to day 84 post immunization. Briefly, purified r6cys A and r6cys B (250 ng/well) in 50 µl total volume of carbonate buffer, pH 9.6, were used to coat ELISA plates overnight at 4 ℃. Then, the plates were blocked using coating buffer containing 2% dry skim milk. The plates were then washed three times with 0.01 M phosphate buffer saline (PBS) containing 0.05% tween 20 at pH 7.2 (PBST), and diluted serum samples (1:25) were added to the plates in duplicate. Plates were incubated for 1 h at 37 ℃, washed as before. Plates were incubated with secondary antibody [goat anti-bovine immunoglobulin G-horseradish peroxidase (IgG-HRP) conjugate (KPL)] at 1:5000 or mouse anti-bovine IgG1-HRP (BioRad) or mouse anti-bovine IgG2-HRP (BioRad) at 1:250 for 1 h at 37 ℃ and washed three times as before. The color reaction was developed by adding 100 μl/well of SuperBlue-TMB Microwell peroxidase substrate (1-Component) (KPL) at 37 ℃. The colorimetric reaction was stopped after 10 min with a solution of 0.16 M sulfuric acid, and the optical density (OD) at 450 nm was determined (Spectramax 190 micro plate reader, Molecular Devices, San Jose, CA, USA). An identical ELISA procedure was utilized for the titration of bovine serum collected after the last immunization (84 dpi) with dilutions including 1:25, 1:50, 1:100, 1:200, 1:400 and 1:800. Secondary antibodies used for titration include goat anti-bovine immunoglobulin G horseradish peroxidase (IgG-HRP) conjugate (KPL) at 1:5000. Statistical analysis was performed to estimate the differences between the two isotypes using two-tailed paired Student's *t*-test.

The presence of bovine anti-6cys A and 6cys B antibodies in tick hemolymph was tested from two groups of ticks fed on immunized and control animals. The first group was collected on the 3rd day post tick dropping (PTD) and the second group on the 5th day PTD. Pooled hemolymph from 100 ticks collected for each group was examined for the presence of bovine anti-6cys A and 6cys B antibodies (total IgG, IgG1 and IgG2) by indirect ELISA, as described above. Each hemolymph group was diluted 1:2 in coating buffer for running the assays. Serum samples collected from immunized and control animals after last immunization were used as a positive control for these assays.

Western blot analysis of native 6cys A and 6cys B proteins was performed as described previously [18], using parasite extracts derived from *B. bovis* parasites induced *in vitro* to form sexual stages and *B. bovis* parasites cultured *in vitro* without sexual stage induction. Parasite extracts were prepared according to Hussein et al. [18]; each extract was electrophoresed in an SDS-PAGE [4–20% Mini-PROTEAN® TGX™ Precast Gels (Bio-Rad)], and proteins were transferred to nitrocellulose membranes. The membranes were blocked with bovine serum albumin (5%) for 1 h and probed with bovine serum from control and immunized animals (serum sample collected before immunization and after the last immunization). The monoclonal antibody (at 1:25 dilution) as a positive control (BABB75) reacted with the ~ 60 kDa RAP-1 [19, 20], Tryp mAb, as a negative control [21]. Membranes were incubated at room temperature (RT) for 1 h and washed three times with PBST. Membranes were incubated with goat anti-bovine immunoglobulin G IgG-HRP conjugate (1:5000) (KPL) for 1 h at RT, and immune complex was developed by chemiluminescent HRP antibody detection reagents (KPL, Gaithersburg, MD, USA). The membrane was stripped and blocked with bovine serum albumin (5%) for 1 h to be used again. Protein Size markers (MagicMarkTM XP Western Protein Standard 20–220, Invirogen, Waltham, MA, USA) are indicated on the left and right ends in each membrane.

### Sexual stage parasite induction inhibition assay

The *in vitro* cultured T2Bo *B. bovis* parasites were used for sexual stage parasite induction, as previously described [18]. The sexual stage induction system was used to test the inhibitory activity of sera from the r6cys A and r6cys B immunized animals and from the rabbit anti-6cys peptide antibodies [12] as described in Additional file 1: Table S1. Pre-immune bovine and rabbit sera were used as negative controls for this experiment. All sera tested (Additional file 1: Table S1) were heat inactivated for 30 min at 56 °C and diluted 1:2 in culture media followed by incubation with infected RBC at 37 °C for 30 min in 96-well plates before induction. Xanthurenic acid was then added to each induced well (100 mM final concentration). The non-induced controls received an equivalent amount of culture media instead of xanthurenic acid. The plates were incubated at 28 °C overnight. Cultures were then examined by preparing thin films on glass slides that were stained by Giemsa and analyzed by light microscopy (100× magnification) to determine the numbers of sexual stage parasites by visual inspection and calculate the parasitemia. Two parameters were then determined for each sample: (1) percentage of intra-erythrocytic parasites and (2) percentage of extra-erythrocytic sexual stage parasites. Sample identification and treatments performed in the sexual stage parasite induction inhibition assay are shown in Additional file 1: Table S1.

### Reactivity of bovine 6cys sera against synthetic 6cys peptide cocktail

ELISA was performed using peptide-coated plates to analyze sera from immunized and control calves. Peptides 6cys A1, A2 and A3 and 6cys B1 and B2 [12] were used to coat ELISA plates. Each peptide was diluted as 1 mg/ml of 1× coating buffer, as a stock solution, and then 1:50 in 1× coating buffer as a working solution. Bovine polyclonal anti-6cys A and 6cys B serum was added as described in the serological analysis section. The polyclonal rabbit antibodies against the peptide cocktails and pre-immune rabbit sera were used as positive and negative controls respectively. Schematic representation of the synthetic peptides along 6cys proteins was generated by visualization of protein domain structure software (http://dog.biocuckoo.org).

### Bioinformatics analysis

The presence of putative N-glycosilation sites in the 6cys A and 6cys B proteins was analyzed using the server NetNGlyc (http://www.cbs.dtu.dk/services/NetNGlyc/).

## Results

### Antigenicity analysis of the *B. bovis* 6cys A and 6cys B recombinant proteins

**Fig. 1:**
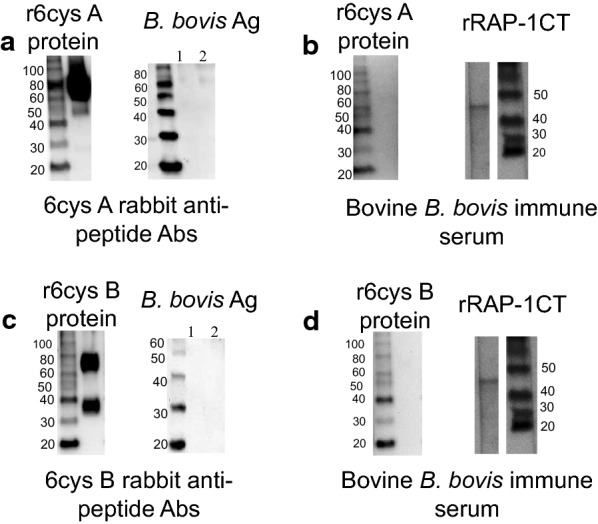
Immunoblot analysis of r6cys A and r6cys B proteins. Reactivity of rabbit polyclonal antibodies against 6cys A and 6cys B peptides, with r6cys A and r6cys B (**a** and **c** left panel), and with *B. bovis* blood cultured lysate antigen (lane 1: uninfected erythrocytes; lane 2: *B. bovis in vitro* culture lysate) (**a** and **c** right panel). Sera from a bovine chronically infected with *B. bovis* recognizes *B. bovis* recombinant protein RAP-1CT (**b** and **d** panels) but does not recognize r6cys A (**b**) and r6cys B (**d**). Protein size markers are indicated on the left and right ends in each panel

Full-length r6cys A and r6cys B proteins containing a His tag were expressed in HEK cells and purified using nickel columns. SDS-PAGE and western blot analysis using anti-His antibodies demonstrated bands at the expected size for r6cys A (66.55 kDa) and r6cys B (67.65 KDa) [12] (Fig. 1 and Additional file 2: Fig. S1). r6cys A and r6cys B proteins were specifically recognized by rabbit antibodies generated against a cocktail of synthetic peptides A and B derived from 6cys A and 6cys B [12] respectively by western blot and ELISA (Fig. 1). The recombinant protein 6cys B includes a protein with the expected size and a lower extra band. This pattern is likely due to the presence of a truncated protein reactive with the antibody in the purified protein fraction. It is possible that this truncated product resulted from earlier termination of translation of the r6cys B due to either the presence of a stop codon in these mRNAs or the presence of truncated mRNA in the HEK cells used for recombinant protein expression.

In addition, bovine sera collected from a calf chronically infected with *B. bovis* did not recognize the r6cys A and r6cys B proteins, while recognizing the positive control, rRAP-1CT protein [15] (Fig. 1b and d). These results suggest that the 6cys A and 6cys B proteins are either not expressed or not immunogenic during *B. bovis* infection in calves.

### Antibody responses in calves immunized with r6cys A and r6cys B proteins

**Fig. 2:**
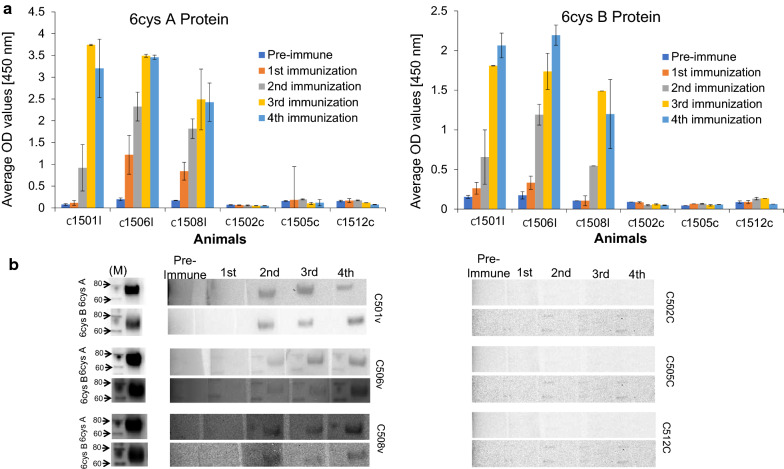
Analysis of the immune responses in cattle immunized with r6cys A and r6cys B. **a** ELISA analysis for the pre-immune and immune serum after immunization with the r6cys A and r6cys B proteins. Y axis represents optical density readings of the ELISA at 450 nm and the X axis the calf numbers; I: immunized; c: control. **b** Western blot analysis of r6cys A and r6cys B protein with pre- and immune serum from the two experimental animal groups taken at different times after immunization

Three calves (C1501, C1506 and C1508) were immunized with purified r6cys A and r6cys B proteins (50 µg each), while three control calves (C1502, C1505 and C1512) were injected with adjuvant only. All immunized animals developed significant levels of antibodies against r6cys A and r6cys B proteins, as detected by ELISA (Fig. 2a). The anti-r6cys A and r6cys B antibody titers reached a plateau between the third and fourth immunizations (Fig. 2a) in all three experimental animals. Consistently, antibodies in sera from all three immunized animals recognized r6cys A and r6cys B proteins by western blotting (Fig. 2b). Thus, two distinct serological analytical methods consistently indicate that immunization with r6cys A and r6cys B elicited antibodies that strongly recognize both r6cys A and r6cys B antigens. Titration of total anti-6cys A and 6cys B antibodies of all experimental calves performed right before the tick challenge in an ELISA is shown in Additional file 5: Fig. S4. Also, immunized animals generated a higher titer of antibodies against the r6cys A compared to the r6cys B antigens. Antibody subclass titers against the r6cys A and r6cys B proteins were also determined by ELISA. There were significantly higher levels (*P* < 0.0053) of IgG2 specific for r6cys A than IgG1, but no significant differences were observed in IgG1 and IgG2 specific for the r6cys B protein (not shown).

### Effect of the immunization on the development of *B. bovis* tick stages upon challenge

**Fig. 3:**
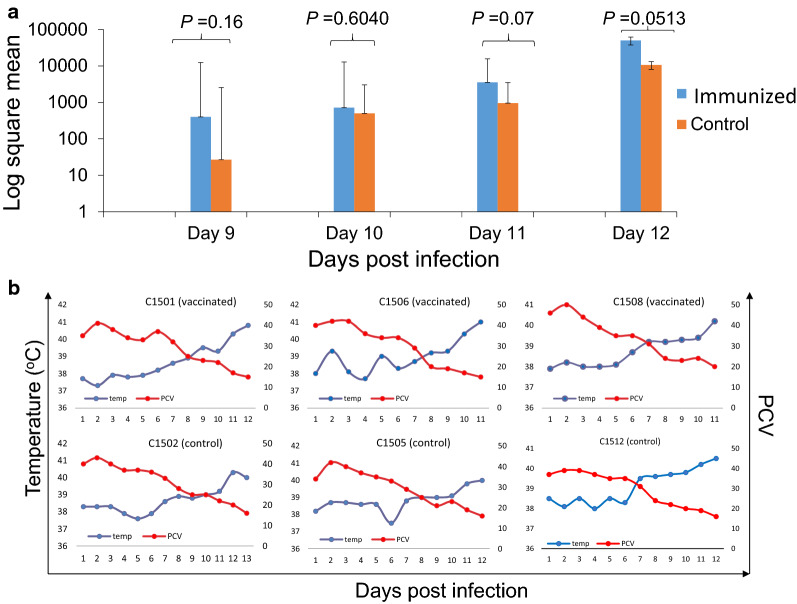
Parasitemia (determined by DNA analysis) and clinical parameters (PCV reduction and temperature rise) in immunized and control animals following *B. bovis* challenge. **a**: Quantitative PCR analysis performed on DNA extracted from blood samples of immunized and control animals at days 9–12 post-challenge. The *P* value for the qPCR data was > 0.05 indicating that there was no significant difference among the samples. **b**: Measurements of temperature and PCV of experimental animals in the two groups from day 1 until day 12 post-infection [dpi]

Immunized and control calves were exposed to *R. microplus* larvae 21 days after the last immunization. Then, all animals were challenged with virulent T2Bo *B. bovis* using the IV route, 12 days after tick applications. All animals were analyzed daily for the presence of *B. bovis* DNA in peripheral blood using conventional PCR and qPCR until day 11 after *B. bovis* challenge, using a primer set that amplifies sequences in the control *B. bovis* rap-1 gene (Fig. 3a and Additional file 6: Fig. S5). PCR results indicated amplification of the *rap-1* gene sequences only in the later days of the experiment, which represent 11 days after the beginning of the *B. bovis* challenge, except in animal C1508 from the immunized group and C1512 from the control group that also displayed PCR-positive dtetection of parasite DNA at 10 dpi. The *B. bovis* challenge resulted in a dramatic decrease in PCV and an increase in rectal temperature in all animals (Fig. 3b). Calves C1506 and C1508 had to be humanely killed at 11 days post-*B. bovis* challenge because of the severity of clinical signs of bovine babesiosis. No typical intra-erythrocytic parasites were detected in Giemsa-stained blood smears in any animal except for a few ring-shaped forms in a couple of animals during the late stage of the acute infection, a typical finding of bovine babesiosis caused by *B. bovis*. There were no significant differences between the immunized and control animals as estimated by qPCR in the last 4 days of the experiment (12 dpi) as shown in Fig. 3a (*P* > 0.05). Overall, the data indicate no differences in clinical signs between immunized and control animals following *B. bovis* challenge confirming that the immunization did not have any significant impact on blood-stage parasites.

We then examined the presence of *B. bovis* kinetes in tick hemolymph from fully engorged ticks collected from immunized and control animals post-tick larvae application. Hemolymph was collected and analyzed from 48 engorged dropped ticks per day for 5 consecutive days. The numbers of kinetes counted per each day are presented in Table 1, and the infectivity rates with the calculated *P* values for the average infectivity rate per tick group are shown in Additional file 7: Fig. S6A. There were no significant differences observed between the average infectivity rates as calculated by *t*-test (*P* > 0.05). In addition, qPCR analysis was performed for the detection of *B. bovis* in hemolymph for ticks that dropped on days 4 and 5 (Group 4 and 5) (Additional file 7: Fig. S6B).

**Table 1:** Percentage infectivity ratios of ticks derived from immunized and control calves

Infectivity ratio (%)						
	Immunized animals			Control animals		
	C1501	C1506	C1508	C1502	C1505	C1512
Day3	0 (0/48)	2.083 (1/48)	4.166 (2/48)	0 (0/48)	6.25 (3/48)	0 (0/48)
Day4	14.583 (7/48)	4.166 (2/48)	2.083 (1/48)	0 (0/48)	20.83 (10/48)	0 (0/48)
Day5	31.25 (15/48)	12.5 (6/48)	12.5 (6/48)	8.33 (4/48)	6.25 (3/48)	2.083 (1/48)

The values were calculated by dividing the total number of kinetes counted by the number of ticks dropped per day from each animal

No significant differences were found in the number of kinetes circulating in the hemolymph among ticks feeding on immunized and control animals (*P* > 0.05), suggesting that immunization with r6cys A and r6cys B antigens had no significant effect on the development of sexual stages and sexual reproduction of the parasite in the midgut of the ticks. Interestingly, we were able to detect anti-6cys A and 6cys B antibodies in the hemolymph of ticks that fed on the three immunized animals (Additional file 8: Fig. S7). Antibody subclass analysis (IgG1 and IgG2 specific for r6cys A) performed on these antibodies in hemolymph demonstrated that IgG2 was present in a higher level compared to IgG1 (Additional file 8: Fig. S7).

The effect of r6cys A and r6cys B immunization on tick weight and tick egg production was also analyzed by weighing collected ticks and pooled eggs. No significant difference was found in the average tick weight (*P* = 0.3, *n* = 96 ticks/animal) between the groups of ticks collected from control and immunized cattle (Additional file 9: Fig. S8A). In addition, no significant differences on the egg mass were detected among ticks developing either on the immunized or control calves (*P* = 1).

We next analyzed the *B. bovis* infection rate in larvae derived from both test groups using qPCR. The experimental design of this study is shown in Additional file 4: Fig. S3 and the results in Additional file 9: Fig. S8B). No significant difference (*P* = 0.4 and *P* = 0.9 for non-induced and induced respectively) was found in the rate of *B. bovis* infection in the progeny of the ticks feeding on immunized or control cattle, indicating that immunization with recombinant 6cysA and 6cysB proteins did not impair transovarial transmission of *B. bovis* to the next generation larvae.

### Differential recognition of native 6cys A and 6cys B proteins among bovine 6cys A and 6cys B antibodies and polyclonal rabbit antibodies upon *in vitro* induction of sexual stages

The *in vitro* system for the induction of sexual forms of *B. bovis* developed in synchronization with these studies in our laboratories revealed that, in contrast to blood stage parasites, *in vitro* induced sexual forms of *B. bovis* expressed native 6cys A and 6cys B proteins. We took advantage of this observation to test whether the antibodies generated in cattle against the recombinant 6cys A and 6Cys B proteins are also able to recognize the native versions of these two proteins in western blots. We also compared their pattern of reactivity and neutralization activity with the polyclonal rabbit anti-peptide antibodies against putative B-cell epitopes of these two proteins.

**Fig 4:**
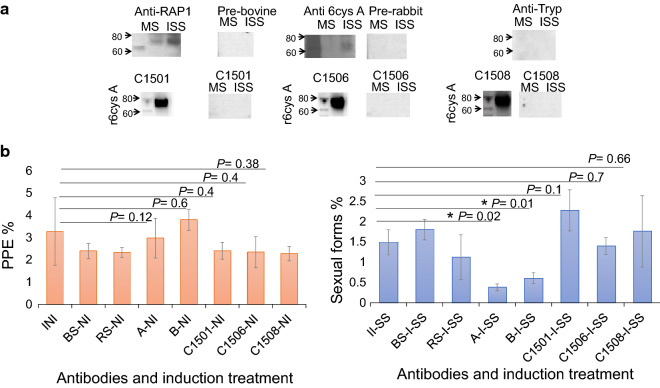
Reactivity of the bovine anti-6cys A and 6cys B polyclonal antibodies against native 6cys proteins. **a** Immunoblot analysis of *B. bovis* merozoite stage (MS) and induced sexual stage (ISS) lysates. Antibodies and serum used are indicated above each lane. **b** Comparison of the *in vitro* neutralization activity of antibodies on the development of sexual stages, performed on the T2Bo *B. bovis* strain. The Y axis represents % of infected RBC “orange bar chart” or the % of sexual forms “blue bar chart.” The X axis represents the rabbit polyclonal antibodies against 6cys A and B peptides and sera from r6cys A and r6cys B immunized cattle. *P* values > 0.05 indicate no significant difference and *P* values < 0.05 indicates there is significant difference. The abbreviations used in the figures are explained in Additional file 1: Table S1

Western blot analysis of *in vitro* induced *B. bovis* sexual stages using rabbit anti-6cys A peptide antibodies demonstrated the recognition of a 66.55 kDa protein, which was not recognized by sera from pre-immune rabbits (Fig. 4a). Such protein was not recognized in an identical analysis performed on non-induced parasites. Also, the monoclonal antibody (mAb) control BABB75 reacted with the ~ 60 kDa RAP-1 antigen in lysates from both induced and non-induced parasites, while the control Tryp mAb showed no reactivity. In contrast, control bovine pre-immune sera and sera from cattle immunized with r6cys A r6cys B proteins did not recognize any protein in either induced or non-induced *B. bovis* lysates (Fig. 4a, Additional file 1: Table S1). However, as described previously, sera from cattle immunized with r6cys A and B proteins did react strongly with r6cys A and r6cys B proteins (Fig. 4a and b and Additional file 3: Table S1). Consistently, polyclonal rabbit serum against 6cys A and 6cys B peptides, but not bovine polyclonal anti-6cys A and 6cys B serum directed against the full-length r6cys A and r6cys B proteins, significantly inhibited sexual stage induction in an *in vitro* assay (*P* < 0.05) (Fig. 4b and Additional file 3: Table S1).

**Fig 5:**
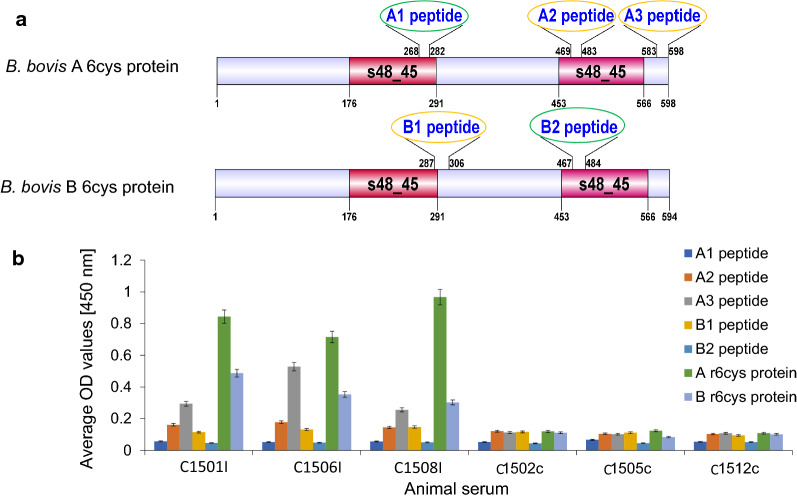
Mapping the reactivity of bovine r6cys A and r6cys B polyclonal antibodies against 6cys A and 6cys B synthetic peptides. **a** Schematic representation of the synthetic peptides selected from the 6cys protein A and B. Peptides A2 and A3 (yellow line), but not A1 (green line) selectively reacted with bovine anti-r6cys A and r6cys B antibodies. Peptide B1 (yellow line), but not B2 (green line) react with bovine anti-r6cys A and r6cys B antibodies. **b** ELISA analysis. The Y axis represents the average of the optical density (OD) values in ELISA at 450 nm. The X axis represents the serum samples tested (samples taken at 84 dpi for both animal groups). Each bar represents reactivity against distinct synthetic peptides and recombinant proteins, as indicated with different color codes shown at the upright of the chart

We then compared the reactivity of the bovine immune sera and rabbit sera with a set of three synthetic peptides derived from the 6cys A protein (peptides A1, A2 and A3) and two synthetic peptides from for the 6cys B protein (peptides B1 and B2) in ELISAs. These sets of peptides represent regions of the 6cys A and 6cys B proteins containing predicted B-cell epitopes that were able to elicit rabbit antibodies that recognize native and recombinant 6cysA and B proteins and inhibit the *in vitro* sexual stage development. The locations of the selected peptides in the 6cys A and 6cys B proteins are represented in Fig. 5a. While the rabbit antibodies specifically recognize all synthetic peptides tested, the antibodies in immunized cattle significantly recognized peptides A3, A2 and B1 (Fig. 5a and b) but failed in reacting with peptides A1 and B2 in the ELISAs (Fig. 5b and Table 2). Interestingly, peptides A1 and B2 represent sequences that are involved in the functional domains s48_45, the defining feature of the 6cys protein family (Fig 5a).

**Table 2:** Serum reactivity with different peptides derived from the *B. bovis* 6cys A and 6cys B proteins

Animals	pA1	pA2	pA3	pB1	pB2	r6cys A	r6cys B
C1501	–	+	+	+	−	+	+
C1506	−	+	+	+	−	+	+
C1508	−	+	+	+	−	+	+
C1502	−	−	−	−	−	−	−
C1505	−	−	−	−	−	−	−
C1512	−	−	−	−	−	−	−

“p” stands for peptide and “r” for recombinant. (– or +): negative or positive reactivity in the ELISA

In summary, while the r6cys proteins A and B used in the immunization of the calves were recognized by rabbit anti-6cys A and 6cys B peptide antibodies as well as the immune sera from immunized animals, the sera from immunized calves failed to recognize peptides A1 and B2 in ELISA (Table 2).

### Detection of N-glycosylation sites in the 6cys proteins

Several putative N-glycosylation sites were predicted in the 6cys A and 6cys B proteins as shown in Additional file 10: Fig. S9. None of these predicted sites in the 6cys A protein were located inside, or in the vicinity, of the regions represented by the 6cys A synthetic peptides. Interestingly, only one of the predicted N-glycosylation sites of the 6cys B protein, 304 NKSI, is present in the B1 peptide. However, the B1 peptide region is recognized by antibodies from immunized bovines, and rabbit antibodies against the B1 peptide were able to inhibit *in vitro* sexual stage development of *B. bovis* parasites, suggesting that the 6cys B protein is not glycosylated in the predicted position or, alternatively, that glycosylation, if it occurred, did not affect the access of antibodies to epitopes in the B1 peptide.

## Discussion

The *B. bovis* 6cys A and 6cys B proteins have been defined as molecular markers of sexual development of *B. bovis* tick stages [12, 13]; as such, they were rational candidates for developing TBVs. However, in this study, we show that antibodies from cattle immunized with a combination of recombinant *B. bovis* 6cys A and 6cys B proteins did not show any inhibitory activity on the formation of sexual forms of *B. bovis* in *in vitro* assays and did not affect the number of kinetes circulating in hemolymph of ticks feeding on immunized animals. This was fully consistent with their inability to recognize native 6cys A and 6cys B from *in vitro* induced sexual forms in immunoblots. It is possible that the failure to recognize the native 6cys A and 6cys B proteins by the sera of immunized animals is due to the lack of conformational space for B-cell epitopes [22] or post-translational modification differences between the recombinant and native proteins. In contrast, rabbit polyclonal antibodies against a cocktail of peptides representing predicted B-cell epitopes, derived from the 6cys A and 6cys B proteins, were able to bind both recombinant and native 6cys A and 6cys B proteins and to inhibit the *in vitro B. bovis* sexual stage production. On the other hand, bovine 6cys A and 6cys B antisera reacted strongly with the r6cys A and r6cys B proteins and had selective reactions against individual peptides present in the peptide cocktails. Bovine 6cys A and 6cys B antisera from immunized animals reacted with peptides A2 and A3 but did not show reactivity with peptide A1. Also, selective reaction was seen with 6cys B peptides as the bovine 6cys A and 6cys B antisera showed reactions only with the B1 peptide but not with the B2 peptides. This is in contrast to the rabbit anti-peptide antisera, which can recognize all these peptides in the assays. Taken together, these data suggest that “transmission-reduction” (TR) requires the formation of antibodies against these regions of the 6cys molecules. Remarkably, both peptides, A1 and B2, not recognized by antibodies from immunized calves, are located inside the predicted functional s48_45 domains of the proteins, as shown in Fig. 5. Altogether, these data strongly suggest that the putative functional regions of these proteins are poorly immunogenic when presented in the context of the full-size protein. This observation can also represent an evolutionary adaptive trait that assures that cattle do not produce antibodies that prevent transmission, since it remains possible that these proteins are still minimally produced by pre-sexual forms of the parasite that may develop in the vertebrate host.

Interestingly, the results of the current study strongly resemble data obtained in four previously published independent immunization experiments using proteins of the 6cys protein family members in *Plasmodium spp.* parasites. These four independent studies also demonstrated a similar inability of antibodies generated against several distinct full-length *Plasmodium* 6cys recombinant proteins to block transmission of the parasite by mosquito vectors. In one study, polyclonal and monoclonal antibodies against *Plasmodium falciparum* Pfs47, a 6cys protein, differentially expressed in gametocytes and female gametes, were also shown to have selective ability to bind different regions of the protein. This protein, containing three 6cys domains, was not able to elicit transmission-blocking activity upon immunization [22]. In contrast, antibodies against a slightly modified version of the Pfs47 D2 domain alone resulted in 78–99% transmission-blocking activity in mice [22]. Comparable results were also obtained testing candidates for a *Plasmodium* TBV based on Pfs230, a protein consisting of 14 cysteine motif (CM) domains. Tachibana et al. [23] have produced and tested whether a single-CM-domain (a total of 14), 2-CM-domain (a total of 7) or 4-CM-domain (a total of 6) has the functional ability to block the transmission. For each fragment, antisera were produced but only antibodies against fragments containing CM domain 1 conferred strong inhibitions in standard membrane feeding assays (SMFAs) while no inhibition was shown with antibodies against any of the other fragments [23].

Finally, similar results were found for the 6cys Pfs48/45 protein, which has important roles in male gamete fertility. Three recombinant fragments of Pfs48/45 were expressed: (1) full-length Pfs48/45, (2) the 6cys domain fragment [C-terminal sequence] and (3) a 6C domain fragment containing two mutations modifying its canonical glycosylation sites. Consistent with our results, no significant transmission-reducing activity was found using the full-size recombinant version of the protein in immunizations. In contrast, significant transmission-reducing activity was found for the 6C domain fragment protein [24]. Furthermore, another study performed on Pfs48/45 found that only the C-terminal domain of Pfs48/45 binds to the effective transmission-blocking antibodies upon mapping with a panel of monoclonal antibodies against the Pfs48/45 protein [25]. These results are consistent with the deglycosylation of the full-length Pfs48/45 recombinant protein, as Pfs48⁄45 is not a glycoprotein in the native hosts but contains predicted glycosylation sites which may be effectively glycosylated during expression in eukaryotic systems, and the sera against this full protein displayed strong inhibition in SMFA [26]. Bioinformatic analysis suggests that the *B. bovis* 6cys A and 6cys B also have putative glycosylation sites, but with the single exception of the 6cys B peptide B1, none of these sites are present in the regions representing the synthetic peptides that can elicit antibodies that inhibit *in vitro* sexual stage transitions. The presence of predicted glycosylation sites suggests that the native 6cys A and 6cys B proteins can be potentially glycosylated, a fact that can result in conformational changes in the protein that may affect their antigenicity. It remains unknown, however, whether glycosylation of these proteins causes conformational effects that may affect the immunogenicity of these two proteins. In addition, it is worth mentioning that *B. bovis,* like *Plasmodium*, probably lacks the full machinery of enzymes required for full N-linked glycosylation [27–29], and as a result, the native 6cys proteins are likely to be different (protein folding, surface glycan profile, stability, etc.) compared to the recombinant 6cys proteins that could be heavily glycosylated during the expression process in HEK 293 cells as an eukaryotic system.

Collectively, the lessons emerging from all of these studies indicate that effective TBV based on 6cys proteins will require the identification of TR epitopes. Antibodies that recognize such TR epitopes are known as functional antibodies. Conversely, other antibodies that lack the ability to affect transmission were defined previously as “non-functional antibodies” [23]. Furthermore, in some cases such non-functional antibodies were also found to enhance transmission [22]. Interestingly, evidence arising from vaccination studies using 6cys proteins as TBV candidates strongly suggests that only certain poorly immunogenic domains of the proteins are able to elicit TB activity. In some cases, these protective epitopes are masked or, alternatively, flanked by non-functionally relevant highly immunogenic regions that only elicit non-functional antibody responses. Here, we compared the *in vitro* sexual stage development inhibitory ability of bovine antibodies specific for full-size recombinant 6cys A and 6cys B proteins with rabbit antibodies specific for predicted regions in 6cys proteins (functionally/antigenically relevant regions of the proteins) and found that only the latter, but not the former, antibodies may function as TR antibodies.

In summary, the results of our study strongly suggest that similar to *Plasmodium* 6cys proteins, the design of *B. bovis* TBV based on 6cys proteins will require first the identification of specific domains of the proteins containing TR epitopes. Experimental evidence collected in this study suggests that the epitopes present in the peptides used to generate the rabbit antibodies may include such protective epitopes. Future research will be focused on these regions of the *B. bovis* 6cys A and 6cys B proteins.

## Conclusions

It is possible that the functionally relevant regions of these proteins have been masked by glycosylation or protein misfolding during the recombinant protein production using the HEK cells or that the immune responses were hampered by highly immunodominant epitopes located nearby, or a combination of factors.

Developing a TBV for *B. bovis* based on 6cys proteins might require further studies to map immunogenic TR epitopes. In addition, a more appropriate recombinant protein expression system able to produce a recombinant protein that more faithfully reproduces the conformation of native *B. bovis* 6cys proteins would be ideal. In fact, that could be achieved, at least in theory, by constitutively expressing His-tagged native 6cys proteins using transfected *Babesia* parasites as a component of a dual-purpose vaccine to prevent acute disease and transmission.

## Supplementary Information

**Additional file 1: Table S1.** Abbreviations used to describe the distinct treatments performed in the *in vitro B. bovis* sexual induction experiment.

**Additional file 2: Fig. S1.** SDS-PAGE and western blot analysis using anti-histidine antibodies reactive with r6cys A and r6cys B proteins. Lane M: protein marker. Lane 1: reducing conditions. Lane 2: non- reducing conditions. Lane P: multiple-tag as positive control. MW of 6cys A: ~ 70 kDa–MW of 6cys B: ~  80 kDa.

**Additional file 3: Fig. S2.** Schematic description of the outline of the in vivo experiments performed in the r6cys A and r6cys B immunization study.

**Additional file 4: Fig. S3.** Schematic representation of the experimental design of larva analysis derived from the two tick groups, immunized and control. Larvae induced under two temperature conditions, 36 ℃ [Induced] and 26 ℃ [Non induced] were analyzed.

**Additional file 5: Fig. S4.** Antibody titrations performed on sera from the six experimental animals against the r6cys A and r6cys B proteins.

**Additional file 6: Fig. S5.** Detection of *B. bovis* DNA by conventional PCR analysis on daily blood samples collected from all animals under experimental study: Immunized animals are C1501, C1506 and C1508. Control animals are C1502, C1505 and C1512. Numbers on top represents days after challenge. Size markers are indicated on the right.

**Additional file 7: Fig. S6.** Tick hemolymph analysis. A: 48 hemolymph samples were analyzed for the presence of kinetes by light microscopy. The numbers of kinete per sample were computed and used to calculate kinete infectivity rates per animal. The chart represents averages of the kinete infectivity rate in hemolymph collected from ticks feeding on immunized and control animals. Analysis was performed on ticks that dropped on the 3rd, 4th and 5th days after the onset of droppings. *P* values (> 0.05) indicate there were no significant differences between the two groups. B: Quantitative PCR (qPCR) analysis for the evaluation of *B. bovis* DNA in hemolymph from the two pooled tick groups derived from the immunized and control animals collected on day 4 and 5, as described in A. There was no significant difference between the two tick groups of tested animals.

**Additional file 8: Fig. S7.** Comparisons of anti-r6cys A and r6cys B antibodies in tick hemolymph and sera from immunized and control animals by iELISA. A: Bovine antibodies were detected in the sera of all immunized animals, 84 dpi, and in the first and second tick hemolymph groups derived from immunized *vs* control animals. B: Antibody isotype analysis performed on antibodies against r6cys A protein present in hemolymph of ticks compared with immunized animals (84 dpi).

**Additional file 9: Fig. S8.** Analysis of the effect of the immunization of cattle with r6cys A and r6cys B proteins on the development of *B. bovis* in ticks. A. Comparisons of tick body (left chart) and egg weights (right chart) among ticks and eggs derived from immunized and control groups. No significant difference among the groups was found (*P* > 0.05). B. qPCR analysis performed on DNA extracted from temperature-stimulated and non-stimulated larvae derived from the two tick groups (vaccinated or control cattle). No significant difference among the groups was found (*P* > 0.05).

**Additional file 10: Fig. S9. **Bioinformatics analysis for the prediction of N-glycosylation sites in the 6cysA and 6cysB proteins. XP_001610178.1 represents the 6cys A protein, and XP_001610179.1 represents the 6cys B protein.

## Data Availability

Data supporting the conclusions of this article are included within the article and its additional files. The datasets used and/or analyzed during the present study are available from the corresponding author upon reasonable request.
