# Supplementary material for: Assessment of Babesia bovis 6cys A and 6cys B as components of transmission blocking vaccines for babesiosis
Source: Parasit Vectors. 2021 Apr 20;14:210. doi: 10.1186/s13071-021-04712-7 (PMC8056569; doi:10.1186/s13071-021-04712-7)
Supplement: Supplementary file 1 — Additional file 1: Table S1. Abbreviations used to describe the distinct treatments performed in the in vitro B. bovis sexual induction experiment. [file 13071_2021_4712_MOESM1_ESM.docx]

**Table S1.** Abbreviations used to describe the distinct treatments performed in the *in vitro* *B. bovis* sexual induction experiment.

| Abbreviation | | Description | *In vitro* sexual induction | Antibody treatment |
| --- | --- | --- | --- | --- |
|  | INI | Infected erythrocytes Non-Induced | No | No |
|  | II | Infected Induced | Yes |  |
|  | II-SS | Infected Induced Sexual stages  (shapes outside RBS) | Yes |  |
|  | BS-NI | Bovine serum not induced | No | Pre-immune Bovine serum |
|  | BS-I-SS | Bovine serum induced sexual stages | Yes |  |
|  | RS-NI | Rabbit serum not induced | No | Pre-immune Rabbit serum |
|  | RS-I-SS | Rabbit serum induced sexual stages | Yes |  |
|  | A-NI | 6cys A serum- not induced | No | Rabbit polyclonal 6cys A antibodies |
|  | A-I- SS | 6cys A serum - induced sexual stages | Yes |  |
|  | B-NI | 6cys B serum- not induced | No | Rabbit polyclonal 6cys B antibodies |
|  | B-I- SS | 6cys B serum - induced sexual stages | Yes |  |
|  | C1501-NI | C1501serum - not induced | No | C1501 Bovine polyclonal anti 6cys A and B serum |
|  | C1501-I- SS | C1501serum - induced sexual stages | Yes |  |
|  | C1506-NI | C1506 serum - not induced | No | C1506 Bovine polyclonal anti 6cys A and B serum |
|  | C1506-I- SS | C1506 serum - induced sexual stages | Yes |  |
|  | C1508-NI | C1508 serum - not induced | No | C1508 Bovine polyclonal anti 6cys A and B serum |
|  | C1508-I- SS | C1508 serum - induced sexual stages | Yes |  |
