# Supplementary figures and images for: Assessment of Babesia bovis 6cys A and 6cys B as components of transmission blocking vaccines for babesiosis
Source: Parasit Vectors. 2021 Apr 20;14:210. doi: 10.1186/s13071-021-04712-7 (PMC8056569; doi:10.1186/s13071-021-04712-7)

## Slide 1
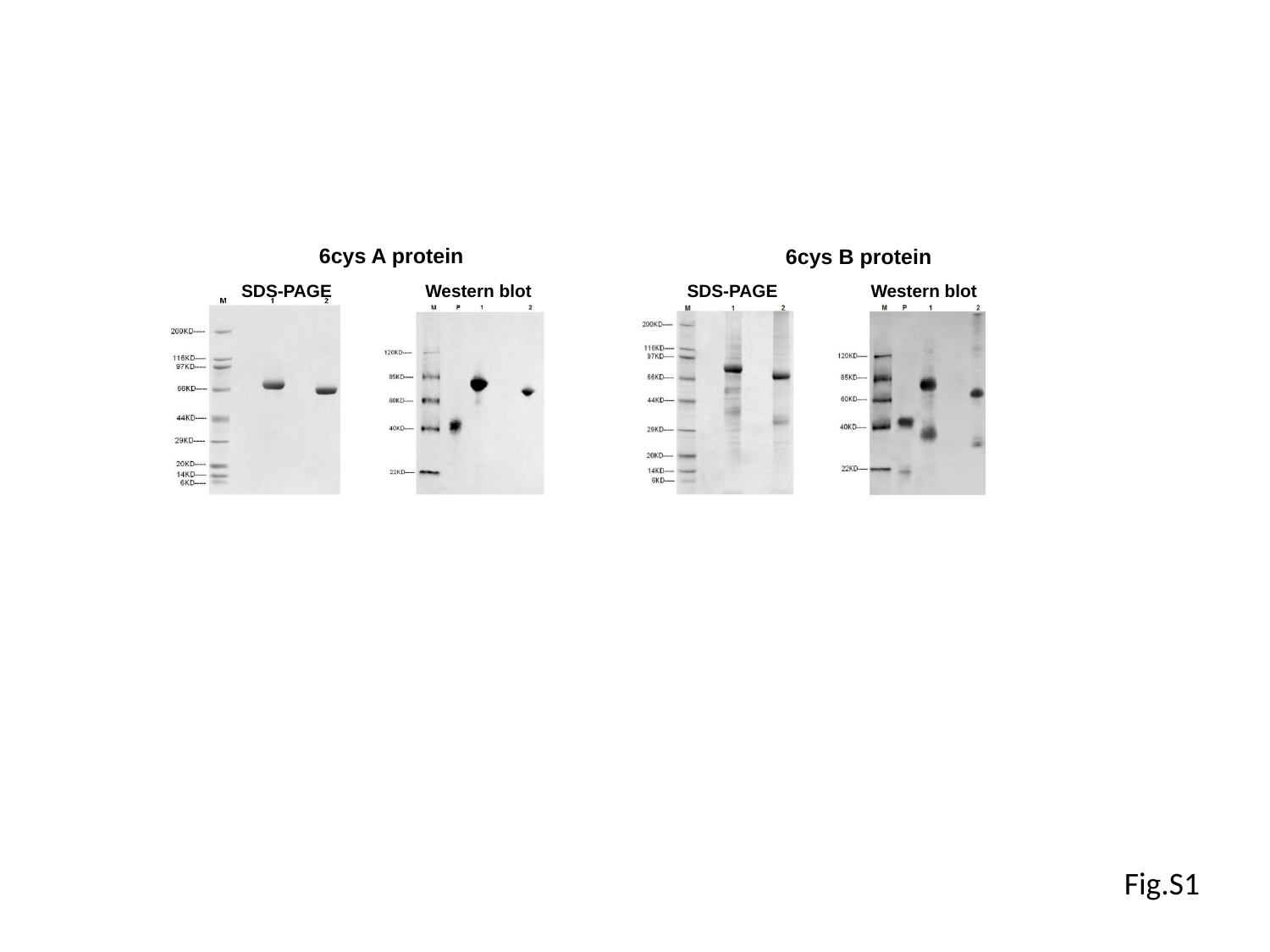

6cys A protein
6cys B protein
 SDS-PAGE
Western blot
 SDS-PAGE
 Western blot
Fig.S1

Supplement: Supplementary file 2 — Additional file 2: Fig. S1. SDS-PAGE and western blot analysis using anti-histidine antibodies reactive with r6cys A and r6cys B proteins. Lane M: protein marker. Lane 1: reducing conditions. Lane 2: non- reducing conditions. Lane P: multiple-tag as positive control. MW of 6cys A: ~ 70 kDa–MW of 6cys B: ~ 80 kDa. [file 13071_2021_4712_MOESM2_ESM.pptx]

## Slide 1
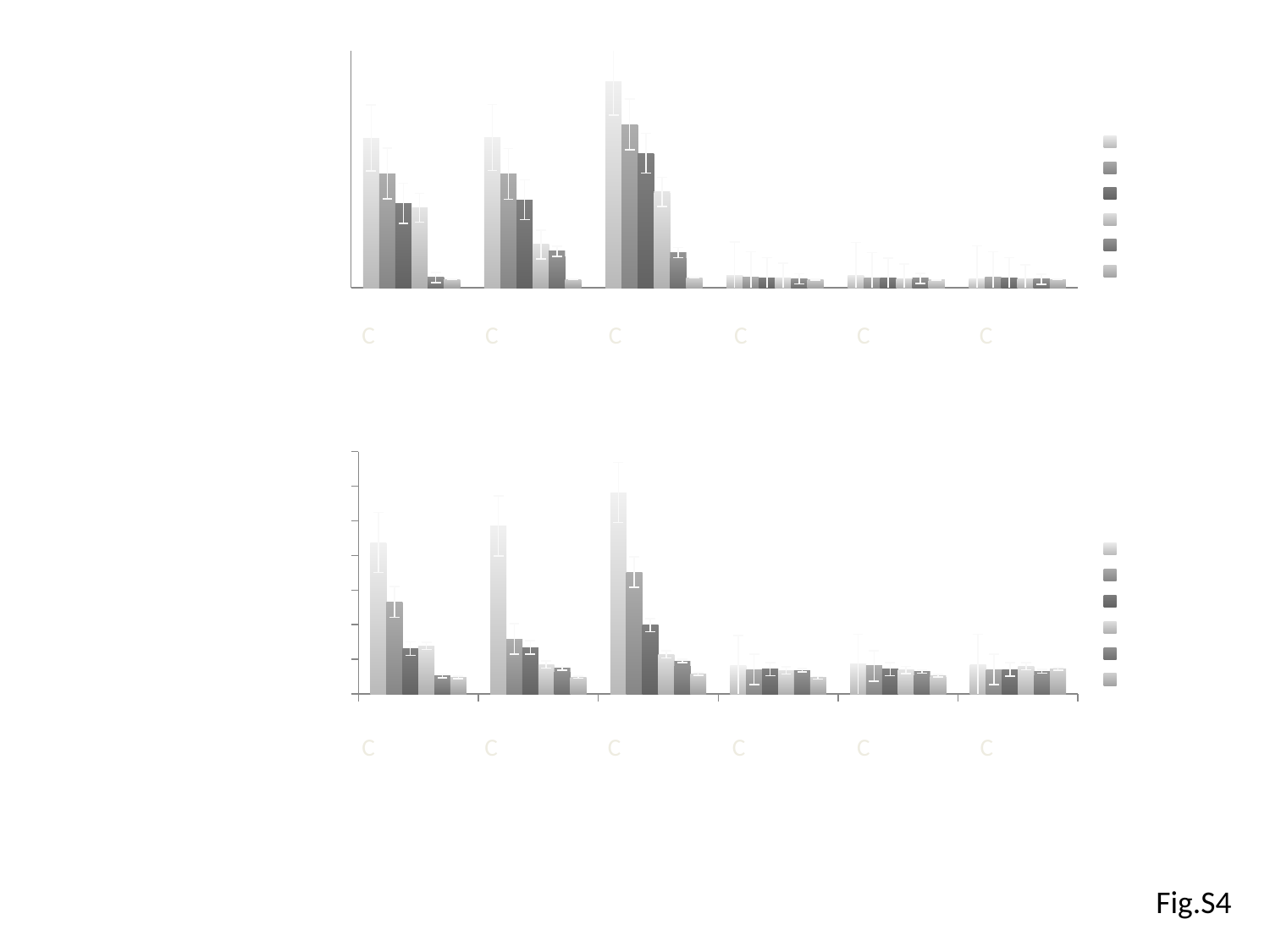

### Chart
| Category | 25 | 50 | 100 | 200 | 400 | 800 |
|---|---|---|---|---|---|---|
| 1501 | 0.8835 | 0.673 | 0.498 | 0.472 | 0.0605 | 0.049 |
| 1506 | 0.887 | 0.671 | 0.519 | 0.255 | 0.215 | 0.0485 |
| 1508 | 1.2145 | 0.9635 | 0.794 | 0.566 | 0.2065 | 0.0595 |
| 1502 | 0.0735 | 0.063 | 0.0595 | 0.059 | 0.052 | 0.047 |
| 1505 | 0.073 | 0.058 | 0.057 | 0.054 | 0.055 | 0.0455 |
| 1512 | 0.053 | 0.0625 | 0.0585 | 0.05 | 0.05 | 0.0485 |
### Chart
| Category | 25 | 50 | 100 | 200 | 400 | 800 |
|---|---|---|---|---|---|---|
| 1501 | 0.4375 | 0.266 | 0.131 | 0.139 | 0.052 | 0.048 |
| 1506 | 0.4855 | 0.159 | 0.1345 | 0.085 | 0.0745 | 0.0485 |
| 1508 | 0.5815 | 0.352 | 0.1985 | 0.1145 | 0.0955 | 0.058 |
| 1502 | 0.0815 | 0.071 | 0.0715 | 0.068 | 0.0685 | 0.047 |
| 1505 | 0.086 | 0.081 | 0.0715 | 0.069 | 0.065 | 0.053 |
| 1512 | 0.0855 | 0.071 | 0.071 | 0.0805 | 0.066 | 0.072 |C
C
C
C
C
C
C
C
C
C
C
C
Fig.S4

Supplement: Supplementary file 5 — Additional file 5: Fig. S4. Antibody titrations performed on sera from the six experimental animals against the r6cys A and r6cys B proteins. [file 13071_2021_4712_MOESM5_ESM.pptx]

## Slide 1
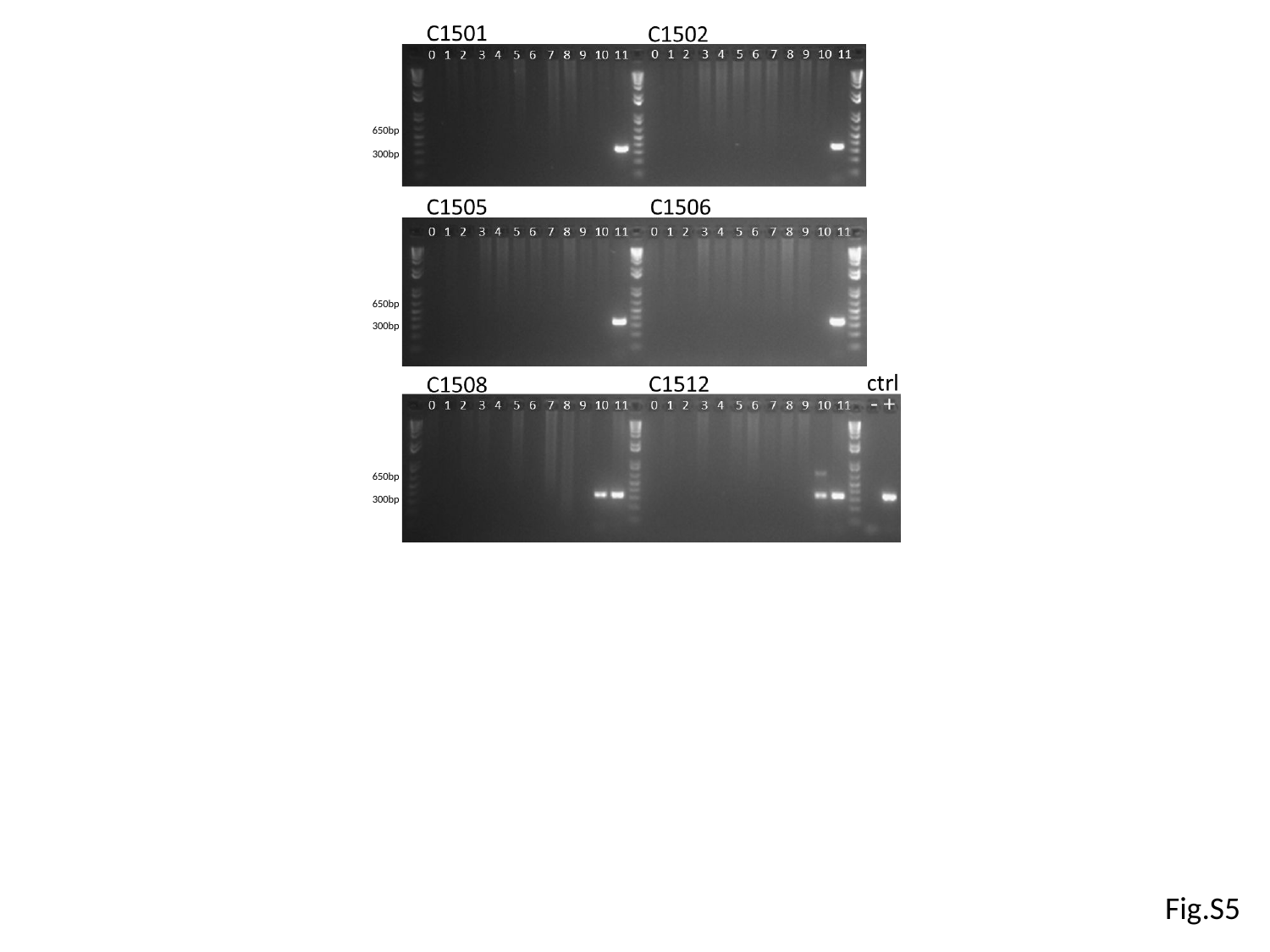

650bp
300bp
650bp
300bp
650bp
300bp
Fig.S5

Supplement: Supplementary file 6 — Additional file 6: Fig. S5. Detection of B. bovis DNA by conventional PCR analysis on daily blood samples collected from all animals under experimental study: Immunized animals are C1501, C1506 and C1508. Control animals are C1502, C1505 and C1512. Numbers on top represents days after challenge. Size markers are indicated on the right. [file 13071_2021_4712_MOESM6_ESM.pptx]

## Slide 1
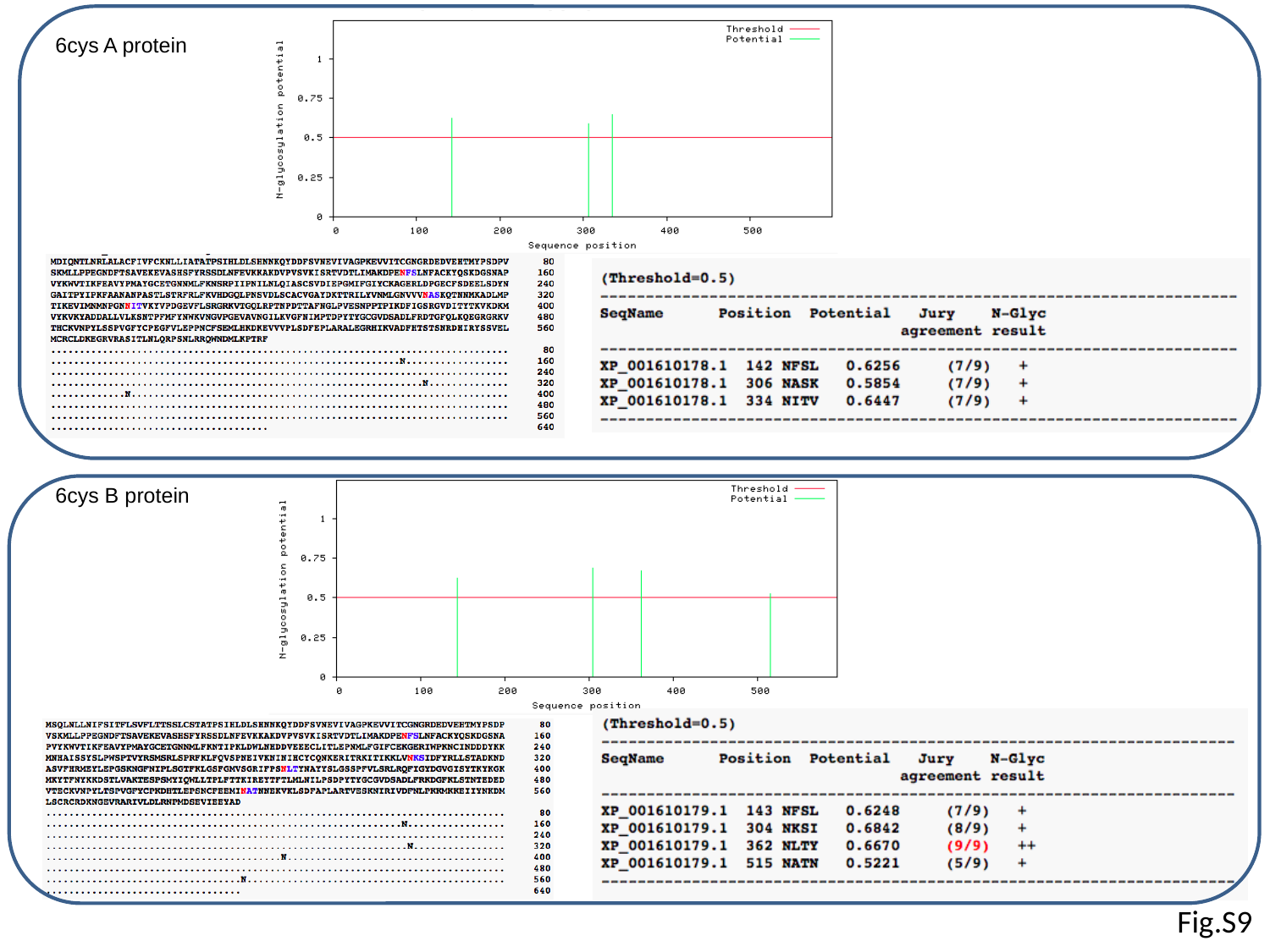

6cys A protein
6cys B protein
Fig.S9

Supplement: Supplementary file 10 — Additional file 10: Fig. S9. Bioinformatics analysis for the prediction of N-glycosylation sites in the 6cysA and 6cysB proteins. XP_001610178.1 represents the 6cys A protein, and XP_001610179.1 represents the 6cys B protein. [file 13071_2021_4712_MOESM10_ESM.pptx]
