# Supplementary material for: Assessment of Babesia bovis 6cys A and 6cys B as components of transmission blocking vaccines for babesiosis
Source: Parasit Vectors. 2021 Apr 20;14:210. doi: 10.1186/s13071-021-04712-7 (PMC8056569; doi:10.1186/s13071-021-04712-7)
Supplement: Supplementary file 3 — Additional file 3: Fig. S2. Schematic description of the outline of the in vivo experiments performed in the r6cys A and r6cys B immunization study. [file 13071_2021_4712_MOESM3_ESM.pptx]

## Slide 1
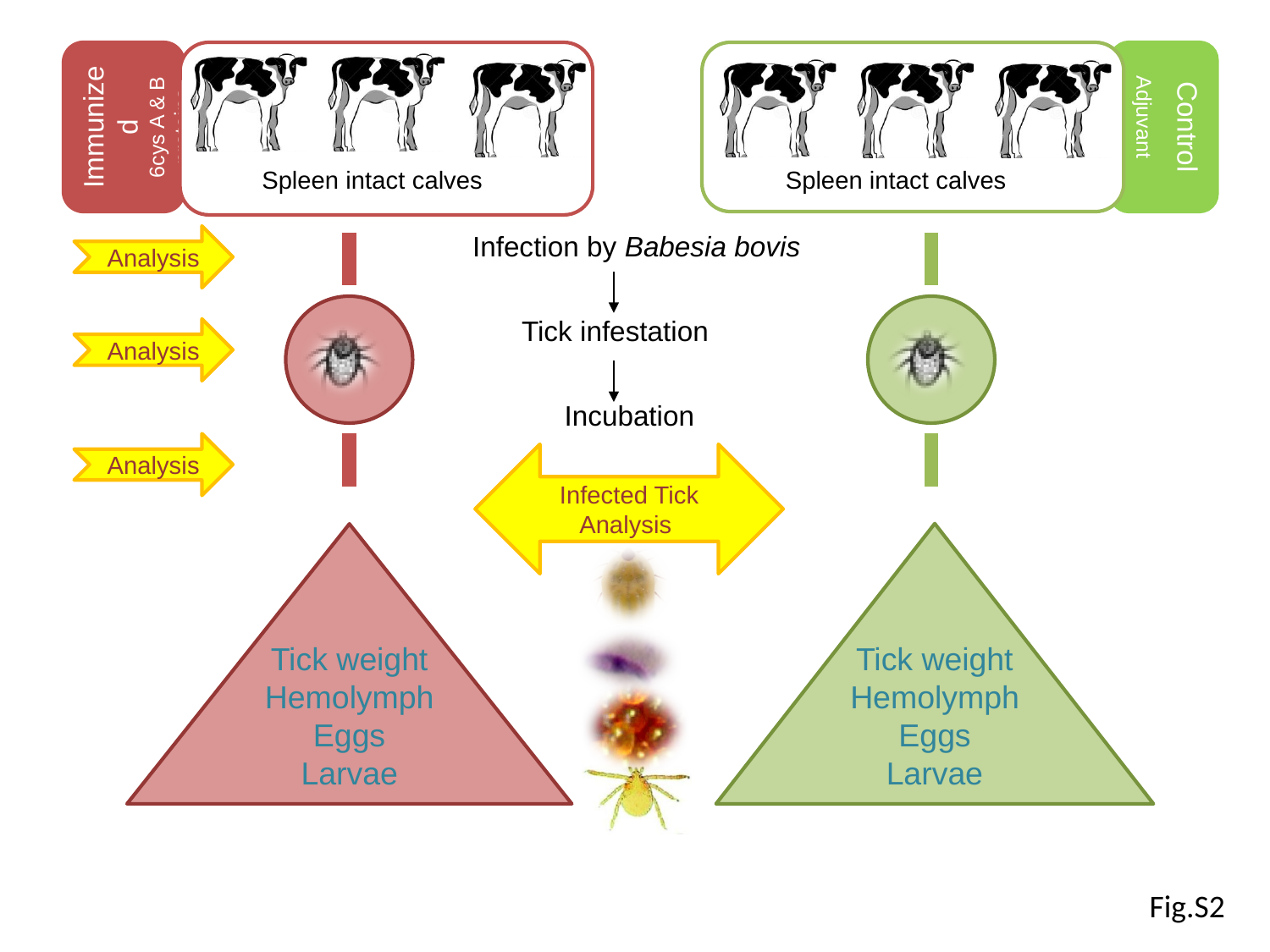

Immunized
6cys A & B proteins
Control
Adjuvant
Spleen intact calves
Spleen intact calves
Infection by Babesia bovis
Analysis
Tick infestation
Analysis
Incubation
Analysis
Infected Tick Analysis
Tick weight
Hemolymph
Eggs
Larvae
Tick weight
Hemolymph
Eggs
Larvae
Fig.S2
