# Supplementary material for: Assessment of Babesia bovis 6cys A and 6cys B as components of transmission blocking vaccines for babesiosis
Source: Parasit Vectors. 2021 Apr 20;14:210. doi: 10.1186/s13071-021-04712-7 (PMC8056569; doi:10.1186/s13071-021-04712-7)
Supplement: Supplementary file 4 — Additional file 4: Fig. S3. Schematic representation of the experimental design of larva analysis derived from the two tick groups, immunized and control. Larvae induced under two temperature conditions, 36 ℃ [Induced] and 26 ℃ [Non induced] were analyzed. [file 13071_2021_4712_MOESM4_ESM.pptx]

## Slide 1
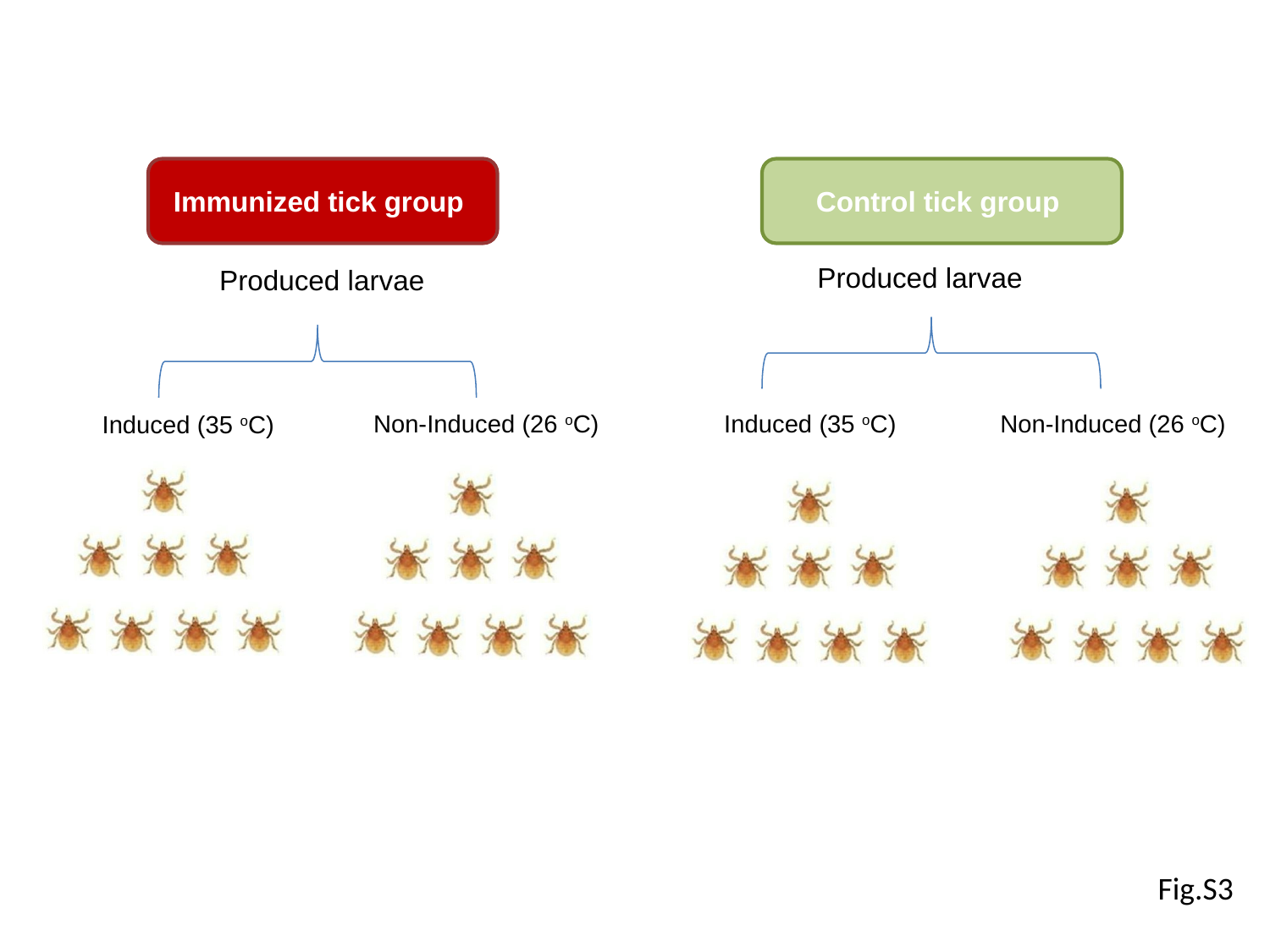

Immunized tick group
Control tick group
Produced larvae
Produced larvae
Non-Induced (26 oC)
Induced (35 oC)
Non-Induced (26 oC)
Induced (35 oC)
Fig.S3
