# Supplementary material for: Assessment of Babesia bovis 6cys A and 6cys B as components of transmission blocking vaccines for babesiosis
Source: Parasit Vectors. 2021 Apr 20;14:210. doi: 10.1186/s13071-021-04712-7 (PMC8056569; doi:10.1186/s13071-021-04712-7)
Supplement: Supplementary file 7 — Additional file 7: Fig. S6. Tick hemolymph analysis. A: 48 hemolymph samples were analyzed for the presence of kinetes by light microscopy. The numbers of kinete per sample were computed and used to calculate kinete infectivity rates per animal. The chart represents averages of the kinete infectivity rate in hemolymph collected from ticks feeding on immunized and control animals. Analysis was performed on ticks that dropped on the 3rd, 4th and 5th days after the onset of droppings. P values (> 0.05) indicate there were no significant differences between the two groups. B: Quantitative PCR (qPCR) analysis for the evaluation of B. bovis DNA in hemolymph from the two pooled tick groups derived from the immunized and control animals collected on day 4 and 5, as described in A. There was no significant difference between the two tick groups of tested animals. [file 13071_2021_4712_MOESM7_ESM.pptx]

## Slide 1
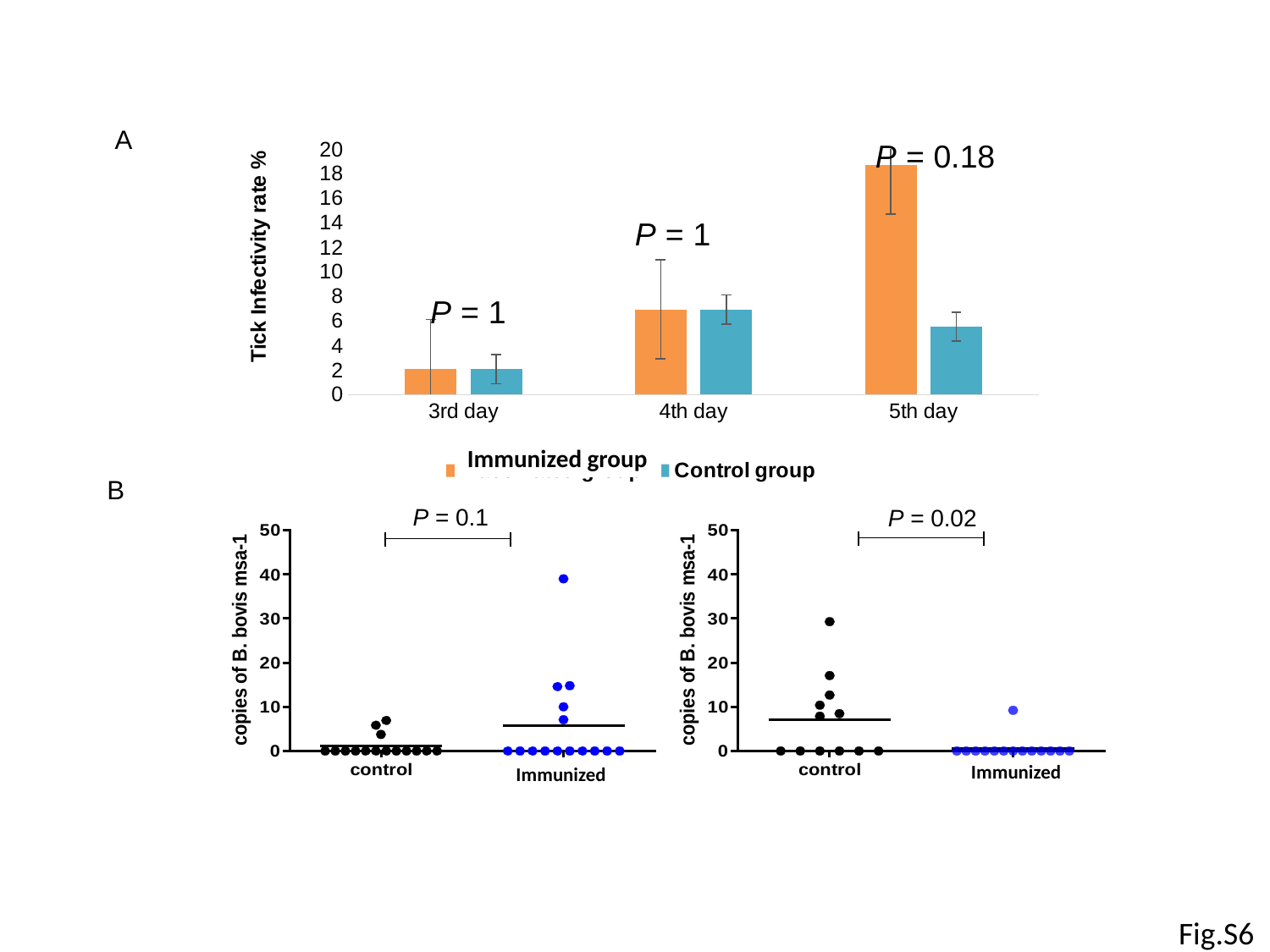

A
### Chart
| Category | Vaccinated group | Control group |
|---|---|---|
| 3rd day | 2.08333333333333 | 2.083333333333333 |
| 4th day | 6.944444444444444 | 6.944444444444444 |
| 5th day | 18.75 | 5.555555555555553 |B
Immunized group
P = 0.1
P = 0.02
Immunized
Immunized
Fig.S6
