# Supplementary material for: Assessment of Babesia bovis 6cys A and 6cys B as components of transmission blocking vaccines for babesiosis
Source: Parasit Vectors. 2021 Apr 20;14:210. doi: 10.1186/s13071-021-04712-7 (PMC8056569; doi:10.1186/s13071-021-04712-7)
Supplement: Supplementary file 8 — Additional file 8: Fig. S7. Comparisons of anti-r6cys A and r6cys B antibodies in tick hemolymph and sera from immunized and control animals by iELISA. A: Bovine antibodies were detected in the sera of all immunized animals, 84 dpi, and in the first and second tick hemolymph groups derived from immunized vs control animals. B: Antibody isotype analysis performed on antibodies against r6cys A protein present in hemolymph of ticks compared with immunized animals (84 dpi). [file 13071_2021_4712_MOESM8_ESM.pptx]

## Slide 1
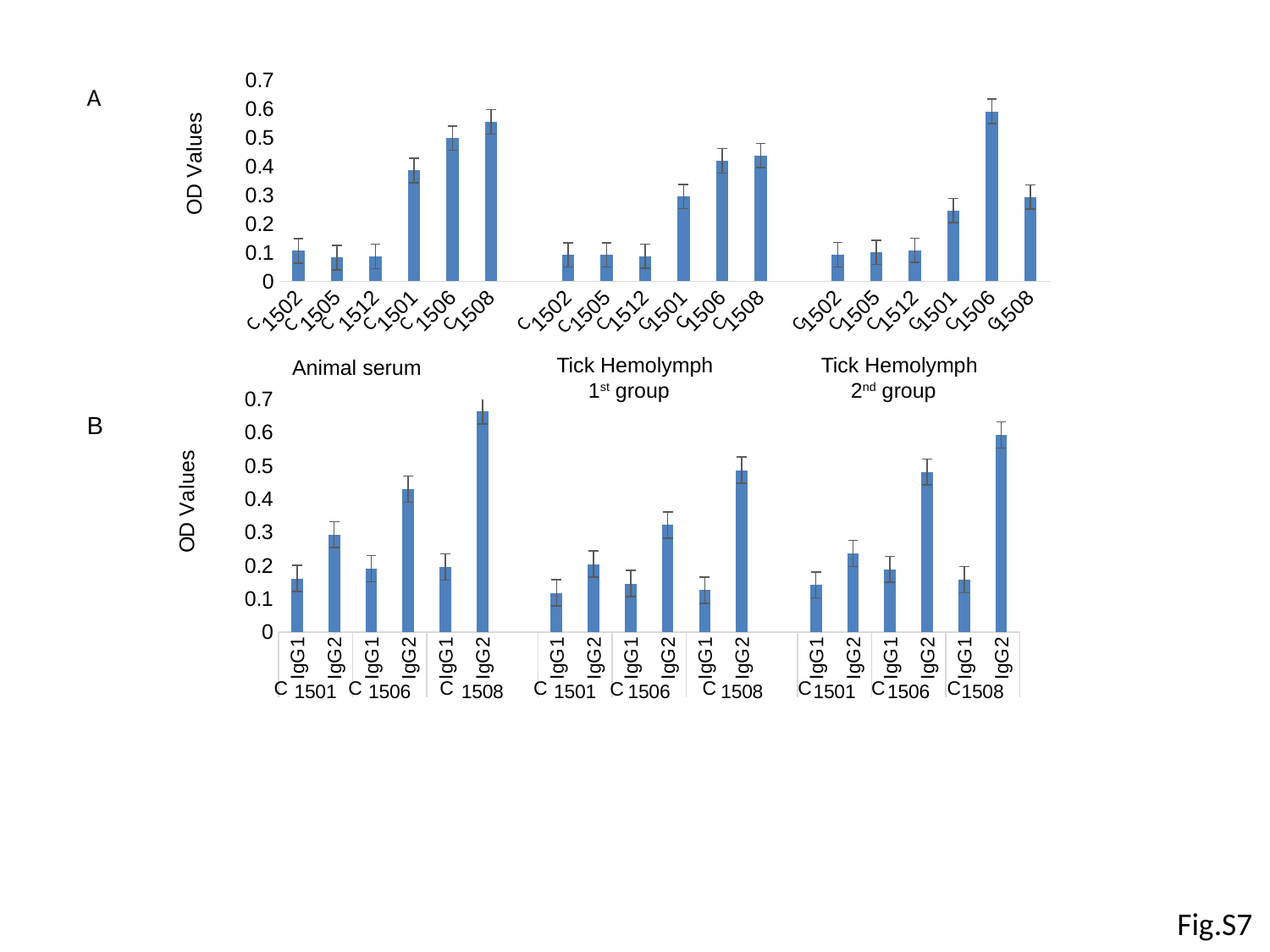

### Chart
| Category | OD values |
|---|---|
| 1502 | 0.1061 |
| 1505 | 0.08235 |
| 1512 | 0.08685 |
| 1501 | 0.3861 |
| 1506 | 0.49825 |
| 1508 | 0.55535 |
| | None |
| 1502 | 0.09225 |
| 1505 | 0.09179999999999999 |
| 1512 | 0.08725 |
| 1501 | 0.29490000000000005 |
| 1506 | 0.41985 |
| 1508 | 0.43815000000000004 |
| | None |
| 1502 | 0.0925 |
| 1505 | 0.1006 |
| 1512 | 0.10845 |
| 1501 | 0.24645 |
| 1506 | 0.59195 |
| 1508 | 0.29364999999999997 |A
Animal serum
Tick Hemolymph
1st group
Tick Hemolymph
2nd group
### Chart
| Category | OD values |
|---|---|
| IgG1 | 0.1616 |
| IgG2 | 0.2938 |
| IgG1 | 0.1915 |
| IgG2 | 0.4306 |
| IgG1 | 0.1966 |
| IgG2 | 0.6657 |
| | None |
| IgG1 | 0.1186 |
| IgG2 | 0.2047 |
| IgG1 | 0.1462 |
| IgG2 | 0.3228 |
| IgG1 | 0.1266 |
| IgG2 | 0.4878 |
| | None |
| IgG1 | 0.1421 |
| IgG2 | 0.2374 |
| IgG1 | 0.1892 |
| IgG2 | 0.4823 |
| IgG1 | 0.1584 |
| IgG2 | 0.5936 |B
C
C
C
C
C
C
C
C
C
C
C
C
C
C
C
C
C
C
C
C
C
C
C
C
C
C
C
Fig.S7
