# Supplementary material for: Assessment of Babesia bovis 6cys A and 6cys B as components of transmission blocking vaccines for babesiosis
Source: Parasit Vectors. 2021 Apr 20;14:210. doi: 10.1186/s13071-021-04712-7 (PMC8056569; doi:10.1186/s13071-021-04712-7)
Supplement: Supplementary file 9 — Additional file 9: Fig. S8. Analysis of the effect of the immunization of cattle with r6cys A and r6cys B proteins on the development of B. bovis in ticks. A. Comparisons of tick body (left chart) and egg weights (right chart) among ticks and eggs derived from immunized and control groups. No significant difference among the groups was found (P > 0.05). B. qPCR analysis performed on DNA extracted from temperature-stimulated and non-stimulated larvae derived from the two tick groups (vaccinated or control cattle). No significant difference among the groups was found (P > 0.05). [file 13071_2021_4712_MOESM9_ESM.pptx]

## Slide 1
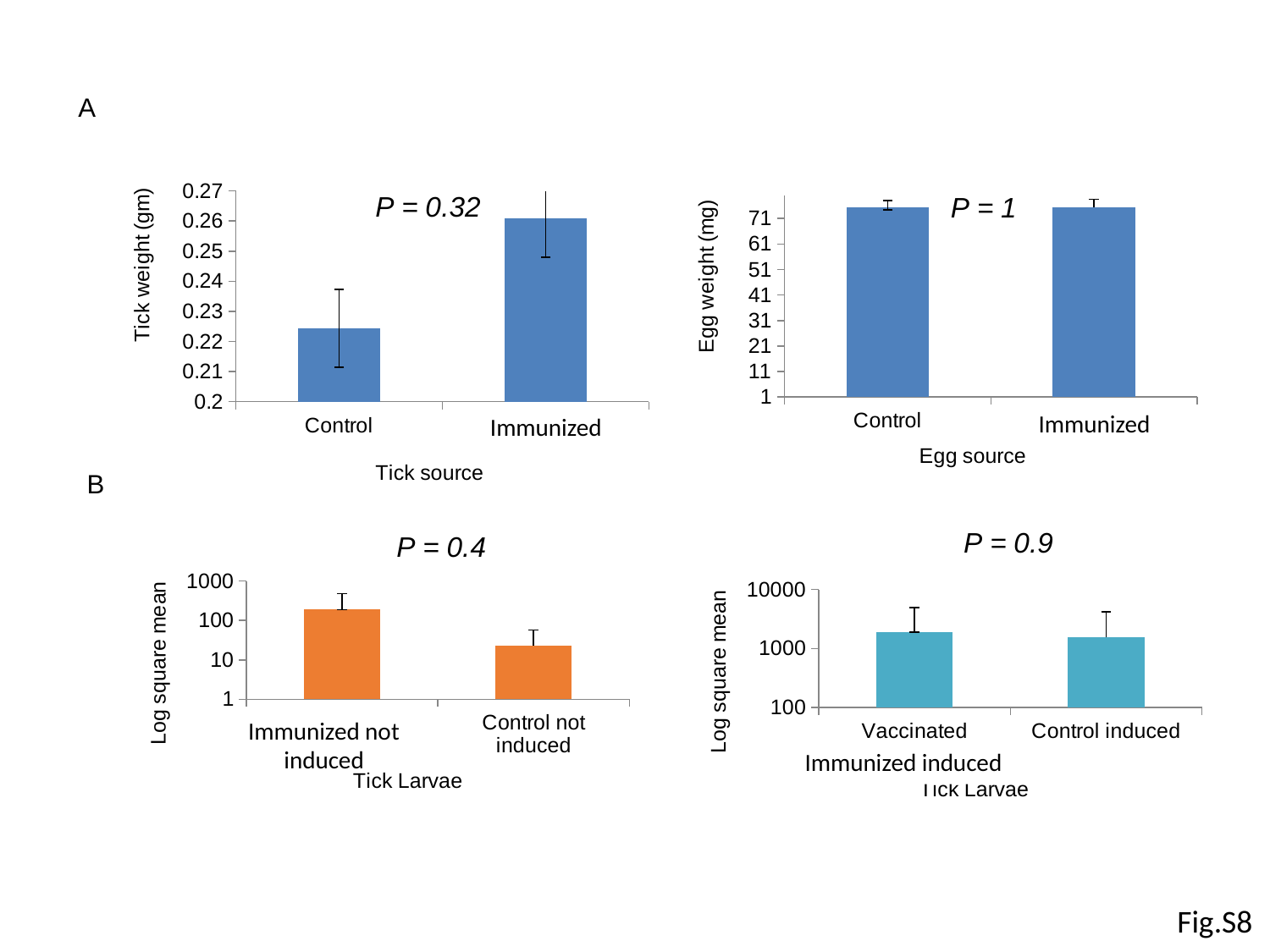

A
### Chart: P = 0.32
| Category | Tick weight (gm) |
|---|---|
| Control | 0.22438733333333336 |
| Vaccinated | 0.2609266666666667 |
### Chart: P = 1
| Category | |
|---|---|
| Control | 75.38289999999999 |
| Vaccinated | 75.5069 |Immunized
Immunized
B
### Chart: P = 0.4
| Category | |
|---|---|
| Vaccinated not induced | 189.84637500000005 |
| Control not induced | 22.792916666666667 |
### Chart: P = 0.9
| Category | |
|---|---|
| Vaccinated induced | 1887.433033333333 |
| Control induced | 1534.9080000000001 |Immunized not induced
Immunized induced
Fig.S8
